# 677. Compression Garment Access and Adherence in a Pediatric Burn Center

**DOI:** 10.1093/jbcr/irag033.558

**Published:** 2026-04-06

**Authors:** Beth Villanueva, Sara O'Rourke, Jessica Gillespie

**Affiliations:** Nationwide Children's Hospital, Columbus, OH; Nationwide Children's Hospital, Columbus, OH; Nationwide Children's Hospital, Columbus, OH

## Abstract

**Introduction:**

Pressure therapy via compression garments is a widely used intervention by burn therapists in the management of hypertrophic scarring following burn injuries. It is recommended that garments apply a defined therapeutic level of compression and are worn consistently for optimal effect. There are many factors that impact adherence to garment use, with unique challenges encountered in the pediatric population. The aim of this project was to identify the success rate of compression garments reaching patients, the speed at which garments are received by patients, reported adherence rates, and barriers to adherence within this pediatric burn center.

**Methods:**

Compression garment receipt and patient adherence were monitored through documentation in the electronic medical record and internal compression garment tracking tool, spanning multiple departments and care settings. Data were evaluated in proportion control charts (p-charts) and x-bar charts using statistical process control methodology to assess both the success rate of garments reaching patients and the level of adherence to garment use. A lag in data was allowed to ensure appropriate time to receive garments, schedule patients, and dispense them.

**Results:**

From May 2024-May 2025, the success rate of compression garments ordered and successfully reaching the patient was 90.7%. On average, garments were received at the institution in 13.4 days and then provided to patients an average of 19.0 days later, giving an average of 32.4 days between ordering the garments and patients receiving. At their follow-up visits, 78.6% of patients reported currently wearing their compression garments. Patient tolerance was cited in 57.1% of responses when patients were no longer wearing their garment.

**Conclusions:**

These baseline data indicated a high success rate (90.7%) in delivering compression garments to pediatric burn patients, with an average turnaround time of 19.0 days from the institution’s receipt to patient delivery. Adherence to garment use was at 78.6%, highlighting both success and ongoing challenges, including patient tolerance. These findings underscore the importance of timely garment delivery and consistent follow-up to support adherence. Further efforts are needed to address barriers specific to the pediatric population to optimize therapeutic outcomes in hypertrophic scar management.

**Applicability of Research to Practice:**

By understanding barriers to time of start of intervention and compression adherence, this may improve the effectiveness of rehabilitation interventions and support future research aimed at optimizing pediatric burn rehabilitation.

**Funding for the study:**

N/A.

